# Rural-Urban Trends for Aortic Stenosis Mortality in the United States, 2008 to 2019

**DOI:** 10.1016/j.jacadv.2023.100617

**Published:** 2023-10-02

**Authors:** Zachary H. Hughes, Michael M. Hammond, Marquita Lewis-Thames, Ranya Sweis, Nilay S. Shah, Sadiya S. Khan

Severe aortic stenosis (AS) confers an approximate mortality rate of 50% within 2 years after the development of symptoms.^1^ With the advent of transcatheter aortic valve replacement (TAVR), older patients with previously prohibitive surgical risk now have a durable therapeutic option. Significant reductions in national age-adjusted mortality rates (AAMRs) due to AS have been observed as the use of TAVR has become more ubiquitous in 2013.^2^ Despite this, TAVR remains a technically intensive procedure requiring a multidisciplinary team of health professionals, which limits accessibility for patients in resource-limited areas and therefore may limit benefits actualized by rural residents.^3^ The American Heart Association released a call to action to address the unique health needs of people living in rural America to reduce and ideally eliminate longstanding health disparities in this population.^4^ To identify opportunities to improve AS outcomes, we compared temporal trends in AS mortality in rural and urban regions of the United States from 2008 to 2019.

We used the Centers for Disease Control and Prevention’s Wide-ranging Online Data for Epidemiologic Research database from 2008 to 2019. AAMRs (standardized to the 2000 U.S. population) and age-specific mortality rates were calculated when AS was the underlying cause of death (International Classification of Disease-10 codes I06.0, I06.2, I35.0, and I35.20). Decedents were categorized by county-level urbanization using the National Center for Health Statistics Urban-Rural Classification Scheme: 4 urban (large central metropolitan, large fringe metropolitan, medium metropolitan, small metropolitan areas) and 2 rural (micropolitan, noncore areas). Rates were stratified by age (<75 and ≥75 years), sex, race, and ethnicity. Temporal trends in rates were characterized by fitting piecewise regression models using Stata version 16 (StataCorp). AAMR ratios and respective 95% CIs represented the number of deaths per 100,000 population in less urbanized counties for every 1 death per 100,000 population in large central metropolitan counties. This study was exempt from review by the Institutional Review Board at Northwestern University because the data are deidentified and publicly available.

**Figure 1:**
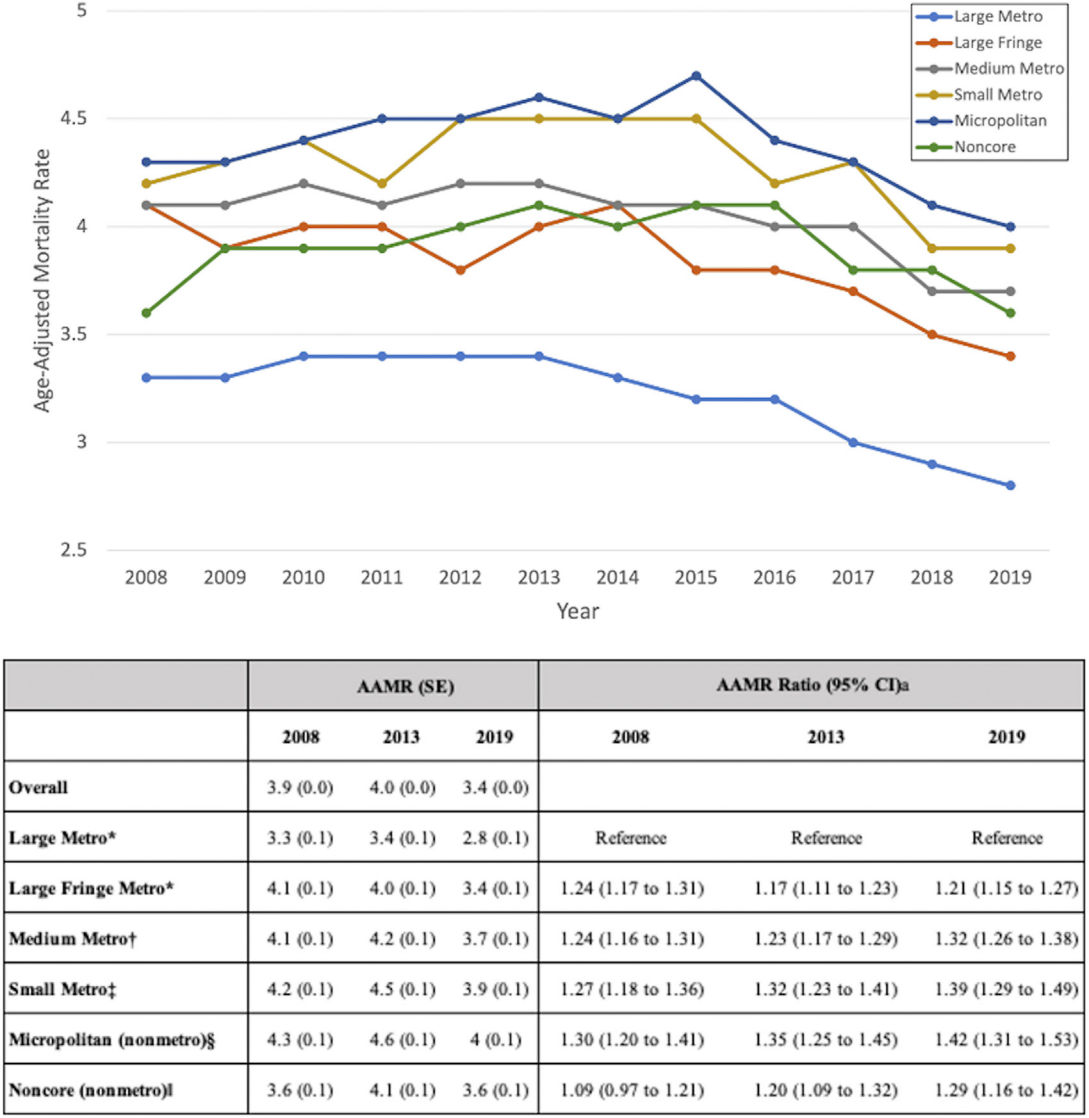
**Age-Adjusted Mortality Rateand Rate Ratios for Aortic Stenosis Mortality in the United States Stratified by County-Level Urbanization, 2008 to 2019**

Between 2008 and 2019, there were 167,808 deaths from AS. AAMRs (standard error) in 2019 were 2.8 (0.1) in large central metropolitan, 3.4 (0.1) in large fringe metropolitan, 3.7 (0.1) in medium metropolitan, 3.9 (0.1) in small metropolitan, 4.0 (0.1) in micropolitan counties, and 3.6 (0.1) in noncore counties per 100,000, respectively. A significant inflection point was observed in overall AAMR trend (Figure 1) with stagnant rates for AS mortality from 2008 to 2013 (regression coefficient for annual change in AAMR per 100,000 population, 0.02; 95% CI: −0.03 to 0.07), followed by a decrease in AAMR from 2013 to 2019 (−0.10; 95% CI: −0.13 to −0.07). ASMRs between large central metropolitan and noncore county decedents ≥75 years were similar in 2013 (51.8 [1.0] and 54.2 [1.9] per 100,000 respectively; AAMR ratio 1.04 [0.97-1.13]) but the difference in ASMRs widened throughout the study period with older large central metropolitan residents having significantly lower rates compared with older noncore county counterparts in 2019 (45.6 [0.9] and 54.1 [1.8] per 100,000, respectively; AAMR ratio 1.19 [95% CI: 1.10-1.28]). There was no change in ASMR for ≥75 years in noncore counties from 2008 to 2019 (54.2 [1.9]-54.1 [1.8] per 100,000). AAMR ratios increased for all urbanization categories compared with large metropolitan from 2013 to 2019 (Figure 1), with the highest increase in AAMR ratio seen in noncore counties (1.09 [95% CI: 0.97-1.21] to 1.29 [95% CI: 1.16-1.42]). In 2019, AAMR ratios were higher in more rural areas. This trend was noted in all age, race, and sex categories.

Between 2008 and 2019, AAMR and ASMR due to AS were higher in rural compared with urban counties. While AS mortality rates decreased for older patients from urban counties, there was no change for those from noncore counties. Rural communities may lack access to specialized cardiovascular care, thereby leading to decreased or delayed disease detection, underutilization of cardiovascular imaging, referrals, and receipt of transcatheter or surgical intervention. Moreover, centers providing TAVR services are located primarily in high-population density areas.^5^ Given the well-established rural-urban disparities in overall cardiovascular disease mortality, AS is of particular importance as a preventable cause of death in the more vulnerable older rural population. Limitations of this study include the use of death certificates, which are subject to misclassification for the cause of death; for instance, more rural areas with less advanced diagnostic capabilities may be less likely to identify AS as a cause of death. However, this data represents the most comprehensive national assessment of mortality statistics. While the decreasing AS AAMR observed after 2013 may be attributed to increasing TAVR use, a causal relationship cannot be inferred. The rural-urban disparity in AS mortality has widened, and urgent interventions are needed targeting optimal cardiovascular care in resource-limited areas.
